# Thermal Stability of TiN Coated Cubic Boron Nitride Powder

**DOI:** 10.3390/ma14071642

**Published:** 2021-03-27

**Authors:** Benjamin Hering, Anne-Kathrin Wolfrum, Tim Gestrich, Mathias Herrmann

**Affiliations:** 1 Institute for Materials Science, Dresden University of Technology, 01062 Dresden, Germany; benjamin.hering@mailbox.tu-dresden.de; 2 Fraunhofer IKTS, Fraunhofer Institute for Ceramic Technologies and Systems, 01277 Dresden, Germany; tim.gestrich@ikts.fraunhofer.de (T.G.); mathias.herrmann@ikts.fraunhofer.de (M.H.)

**Keywords:** cubic boron nitride, cBN, titanium nitride, TiN, coating, thermal stability, hexagonal boron nitride, hBN, phase transformation, microstructure

## Abstract

Wear-resistant, super hard ceramic composites based on cubic boron nitride (cBN) are of great interest to industry. However, cBN is metastable under sintering conditions at normal pressure and converts into the soft hexagonal BN (hBN). Therefore, efforts are being made to avoid this process. Besides short sintering times, the use of coated cBN-particles is a way to minimize this process. Therefore, the thermal stability of TiN coated cBN powders in high purity argon and nitrogen atmospheres up to temperatures of 1600 °C was investigated by thermogravimetry, X-ray phase analysis, scanning electron microscopy and Raman spectroscopy. The TiN coating was prepared by the atomic layer deposition (ALD)-method. The investigations showed that the TiN layer reacts in Ar at T ≥ 1200 °C with the cBN and forms a porous TiB_2_ layer. No reaction takes place in nitrogen up to temperatures of 1600 °C. Nevertheless, the 20 and 50 nm thin coatings also undergo a recrystallization process during heat treatment up to temperatures of 1600 °C.

## 1. Introduction

Due to the increasing need for wear-resistant components, the development of such ceramic composites is of great interest. Possible candidates are diamond or cubic boron nitride (cBN)-based ceramic composites. Because of the metastability of diamond and cBN under normal pressure and higher temperatures, cBN–ceramic and diamond–ceramic matrix composites are mostly produced under high-pressure conditions up to now. This results in restrictions in geometry and costs. Therefore different attempts were carried out to produce cBN-based ceramic composites, to find sintering conditions for manufacturing at low pressure and evaluating the properties of these composites with cubic boron nitride [1,2,3,4,5,6,7,8,9].

New sintering techniques like field-assisted sintering technology (FAST), also known as spark plasma sintering (SPS) or pulsed electric current sintering (PECS), allow a fast densification of these composites. The aim of such fast pressure-assisted densification methods is to prevent the transformation of the cBN into the hexagonal BN modification (hBN), which would otherwise lead to a significant decrease in hardness and wear resistance [10,11].

The stability of cBN is still under discussion. Transformation temperatures of cBN to hBN from 0 K up to 1300 °C were described (boundary of the thermodynamic stability at ambient pressure) [1,12,13,14,15,16,17,18,19,20,21,22]. The detailed review of the literature and the behavior in different ceramic composites [1,21,22,23] reveal, that the transformation temperature must be at least below 1100 °C. The transformation is strongly influenced by kinetic factors. 

Cahill et al. [24] heat-treated three cBN powders with a different particle size at ambient pressure under 1 atm helium for 8 h at temperatures between 1560 and 1660 °C to analyse the kinetics and mechanism of cBN to hBN transformation. The powders with 2–4 µm and 35–45 µm cBN particles showed a start of cBN/hBN transformation at 1580 °C. The cBN powder with <0.5 µm particle sizes had a transformation starting temperature at 1560 °C. Using SEM and TEM, the morphology of the hBN growing showed differences between the various particle sizes, which is attributed to a reduction in nucleation site availability. They concluded that small amounts of impurities or fluxes can lower the starting temperature of the cBN/hBN transformation strongly.

Wolfrum et al. [23] investigated the thermal stability of a pure and a partially oxidized cBN powder at a temperature of 1550 °C. The pure powder showed no evidence of hBN formation whereas the oxidized powder exhibited a significant formation of hBN on the surface of the cBN particles. Additional investigations of the transformation of cBN in different glass- and Si_3_N_4_-matrices proof that the transformation is accelerated by a liquid oxide or oxynitride phase [23]. 

Due to these results [23], Wolfrum [1] analyzed if a titanium coating on cBN particles could suppress the hBN formation during sintering of SiAlON–cBN composites. However, the titanium coating proved to be unstable, reacted into TiN, resulting in a porous interlayer. This layer was not able to prevent the hBN formation during sintering of SiAlON–cBN composites by FAST method. 

The influence of Ni [25,26] and SiO_2_ [27] coatings on the transformation behavior of cBN particles was investigated to avoid the hBN transformation during the SPS-process. At a sintering temperature of 1400 °C no hBN formation could be detected.

Hotta et al. [28] have sintered TiN–cBN-composites using FAST method. The results of the XRD measurements show that no hBN was formed until a sintering temperature of 1650 °C. However, in previous investigations of Al_2_O_3_–cBN-composites a starting temperature of hBN formation at 1400 °C was observed [29]. The starting temperature of 1650 °C was determined for hBN formation in a monolithic cBN body by the same authors. This is the same as for the TiN–cBN-composites [30]. In all TiN–cBN composites sintered in a temperature range of 1600–1800 °C a small amount of TiB_2_ could be found via XRD. 

Kitiwan et al. [31] used FAST for the densification of hBN-TiN composites between 1700 and 2000 °C. Up to 1700 °C no formation of TiB_2_ was observed indicating the high kinetic stability of the metastable hBN–TiN mixture. 

Xie et al. [32] produced TiN–cBN composites with high-pressure high-temperature method (e.g., 1450–1550 °C; 6 GPa). Only traces of TiB_2_ were observed. 

Umer et al. [33] sintered also with high-pressure high-temperature method cBN composites with sol gel coated TiN–cBN powders (details of the sol gel TiN coating process can be found in a previous work of Umer et al. [34]) densified with TiN and Al at 1500 °C and 6 GPa. The XRD pattern showed only traces of TiB_2_. However, the interaction was slightly higher for the coated powder in comparison to the mixture of uncoated cBN and TiN powders. The sol gel TiN coating of cBN powders was not dense [34]. Similar results we found in own investigations concerning sol gel coatings.

With the results of Hotta [28,30] and the comparisons worked out by Wolfrum [1] and Zhang et al. [9] concerning the influence of different matrix materials on the transformation temperature of cBN within the FAST process, it is shown that a TiN-based matrix neither contributes to an acceleration nor to a retardation of hBN formation. Based on this, a dense and homogeneous TiN coating on the cBN particles could lead to the stabilization of the cBN modification during the sintering process using FAST, even with the presence of a liquid phase.

Therefore, in the present work, the stability of titanium nitride (TiN) coatings on cBN particles and its influence on the cBN to hBN-transformation was investigated using thermogravimetric analyses followed by FESEM and XRD analysis and Raman spectroscopy. 

## 2. Materials and Methods

**Figure 1:**
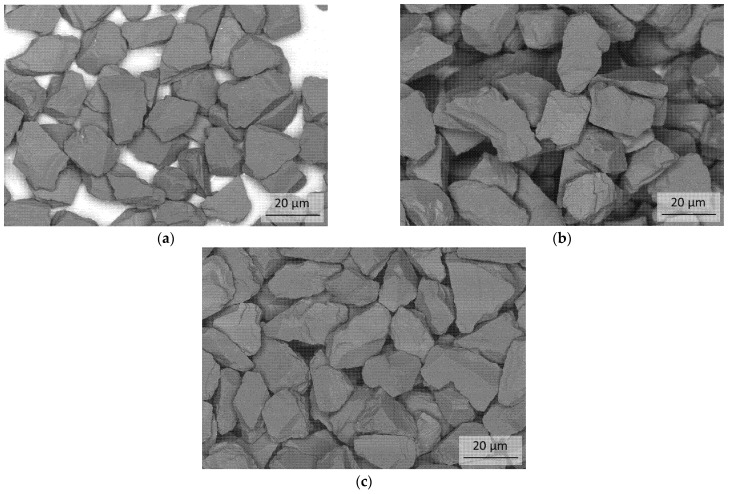
FESEM micrographs of the cBN powders in the initial state (as-received state); (**a**) cBN-B20; (**b**) cBN-B21; (**c**) cBN-C41.

Three cBN powders with an average particle size of 20 µm B20, B21 and C41 from Vollstädt-Diamant GmbH (Seddiner See, Germany) were used. FESEM Micrographs of each powder as received is shown in Figure 1. The TiN coating was prepared with a Fraunhofer IKTS intern developed coating system based on the atomic layer deposition (ALD) on powder B20. For the coating, the as-received powder and a powder heat-treated at 1000 °C in hydrogen atmosphere directly prior to the coating were used. In the further course of the work, the coated powder without pre-treatment will be referred to as BT powder and the coated powder cBN with hydrogen pre-treatment as BTV. Details of the coating methods are explained in [35,36,37,38].

Prior to the analysis of the thermal stability of the different cBN powders, they were analyzed regarding their oxygen content through hot gas extraction (LECO TCH 600, LECO Corporation, St. Joseph, MI 49085, USA) and their metallic impurities by X-ray fluorescence analysis (XRF) (Bruker S8 Tiger, Bruker AXS, Karlsruhe, Germany). The phase content before and after thermal treatment of the powders was analyzed by X-ray diffraction (XRD) using a diffractometer (D8 Advance, Bruker AXS, Karlsruhe, Germany), operated with Cu-Kα radiation with a LynxEye position-sensitive detector (PSD) over a two-theta range of 10–100° 2θ with a step size of 0.02° 2θ. For the qualitative evaluation, the software DIFFRAC.EVA (Bruker AXS, Karlsruhe, Germany) and a PDF (Powder Diffraction File from the International Centre for Diffraction Database 2021) structure database were used. Quantitative evaluation was carried out using the Topas6.0 software (Bruker AXS, Karlsruhe, Germany).

The occurrence of hBN after heat treatment of the powders was analyzed using the Raman spectroscopy as well as the initial cBN powders for comparison. A LabRAM HR800 (Horiba Scientific Ltd., Bensheim, Germany) with a blue laser with a wavelength of 473 nm and an approximate measuring spot size of 0.8 μm was used.

Two field emission scanning electron microscopes (FESEM, Ultra 55 and NVision 40, Zeiss Ltd., Oberkochen, Germany) with an ESB (energy selective backscattered electrons) detector (Zeiss Ltd., Oberkochen, Germany) for material contrast and an SE2 (secondary electron) detector (Zeiss Ltd., Oberkochen, Germany) for topography contrast were used to investigate the microstructure of the TiN coated powders and the thermally treated cBN powders.

For characterizing the TiN coating thickness and their microstructure, TiN coated cBN particles were embedded in epoxy resin and cross-sections were prepared by broad ion beam polishing (BIB) using BalTec RES 101 (Bal-Tec AG, now: Leica Microsystems GmbH, Wetzlar, Germany) [39]. 

The thermal stability of the cBN powders was investigated using thermogravimetric analysis in a STA 429 CD with tungsten furnace (NETZSCH-Gerätebau GmbH, Selb, Germany) and graphite crucibles. A heating rate of 10 K/min up to 1600 °C and 10 L/h argon or nitrogen flow were used. A holding time of 60 min at 1600 °C was applied. The cooling rate was 20 K/min. Only with the tungsten furnace is it possible to achieve the low oxygen partial pressures required for the experiments. The absolute masses of the analyzed powders by TG ranged from approximately 180 to 1000 mg due to the different available masses after coating using the ALD process. The different mass could be also the reason of the slightly different noise of the mass change signal.

The heat treatment temperature was chosen because at 1550 °C uncoated cBN particles showed no transformation in earlier experiments [23]. Therefore, it was decided to increase the temperature to determine the protective effect and stability of the coatings. However, the densification of cBN ceramic matrix composites usually takes place below 1600 °C. Therefore, we choose this temperature for the experiment.

Thermodynamic calculations were carried out using FACTSAGE 8.0 (CRCT, Montreal, QC, Canada and GTT-Technologies, Herzogenrath, Germany) [40]. The database does not contain the data of cBN. Therefore the data in [1,23] were used for cBN.

## 3. Results

### 3.1. Characterization of the Initial cBN Powders

The properties of the powders are given in Table 1. The thickness of the coating depends on the pretreatment. Without hydrogen pretreatment, a thickness of 50 nm was determined by FESEM micrographs of cross-sections of the BT powder. For the BTV powder with hydrogen pretreatment, only a thickness of 20 nm was observed. This could be caused by the different nucleation rate of the TiN coating. The different coating thickness can also be confirmed by results of the quantitative analysis from XRF and XRD, as only a TiN mass fraction of approximately 1.3 wt. % was determined for the BTV powder and approximately 2.3 wt. % for the BT powder.

**Figure 2:**
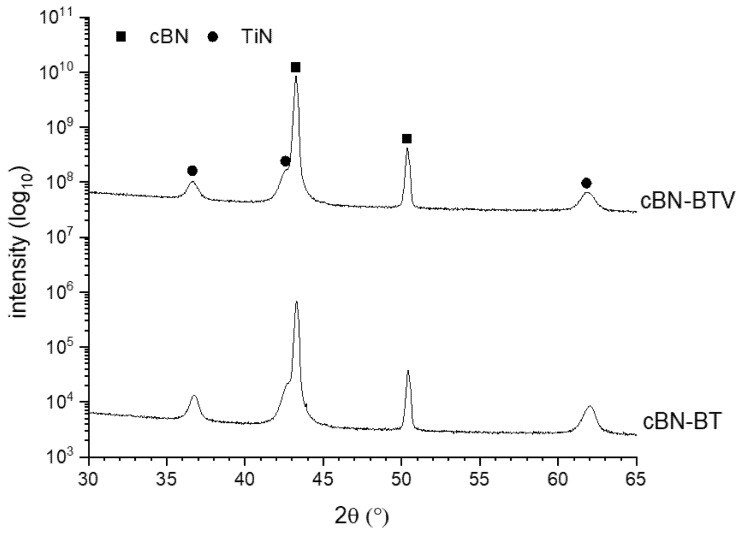
XRD-pattern of the TiN coated cBN powders BT and BTV.

The XRD pattern in Figure 2 of the starting powders reveals besides cBN (ICDD card: 00-035-1365) the TiN (ICDD card: 01-087-0627) formation.

**Figure 3:**
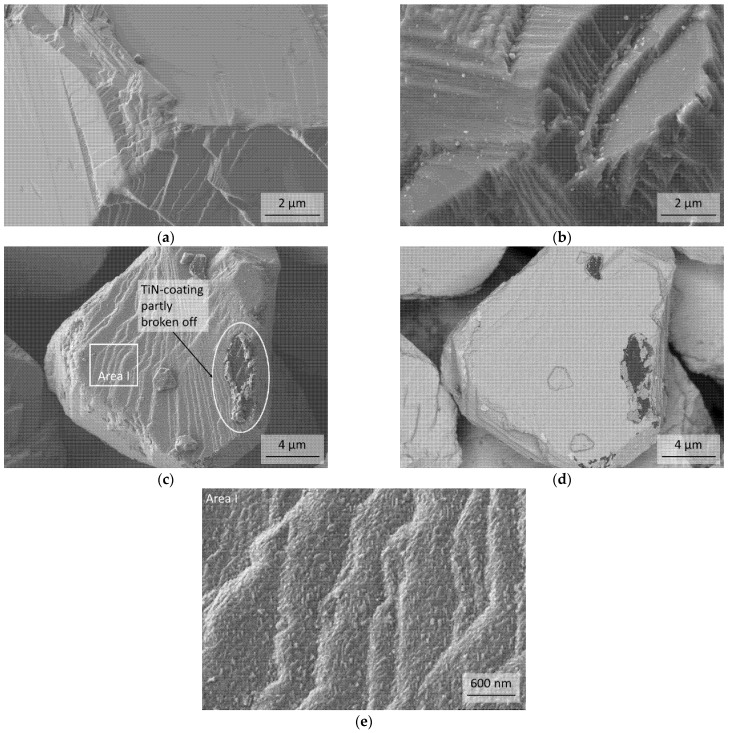
FESEM micrographs of uncoated cBN particles B21 (**a**) and coated with TiN BT (**b**–**e**) before the heat treatment. (**d**) same area as (**c**) with material contrast (energy selective backscattered electrons (ESB)-detector); (**e**) is a section (Area I) of micrograph (**c**) to reveal the microstructure of the TiN coating in more detail.

The FESEM micrograph of Figure 3a shows the surface of a cBN particle of the uncoated B21 powder and Figure 3b–e show different TiN coated particles of the BT powder before the heat treatment at 1600 °C. No impurities at the surface of the uncoated cBN B21 could be found. For the TiN coated powder BT it can be proven, that the cBN particles are mostly completely coated with TiN. Only a few particles exhibit a partly damaged coating as it is shown in Figure 3c with topography contrast and Figure 3d with material contrast respectively, probably due to contact with another cBN particle during the coating process. The microstructure of the TiN coating in more detail is demonstrated in Figure 3e.

**Figure 4:**
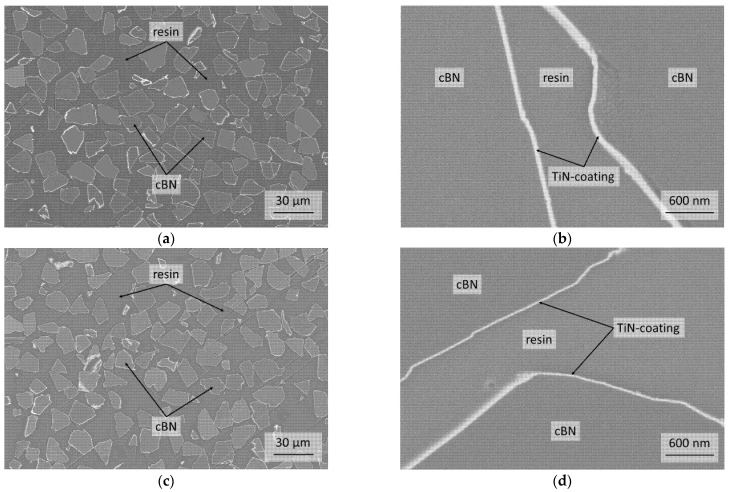
Cross-sections via FESEM (material contrast) of the (**a**,**b**) TiN-coated BT and (**c**,**d**) BTV powder.

Figure 4 shows the cross-sections of the TiN coated cBN powders BT and BTV. Both powders exhibit a dense and homogeneous TiN layer on the cBN particles. Every cBN particle could be coated with TiN, while a few particles show thicker coatings (see Figure 4a,c). It was observed that the BT powder has a thicker TiN coating (approximately 50 nm) than the BTV powder (approximately 20 nm) (see Table 1).

### 3.2. Thermal Stability of the cBN Powders

**Figure 5:**
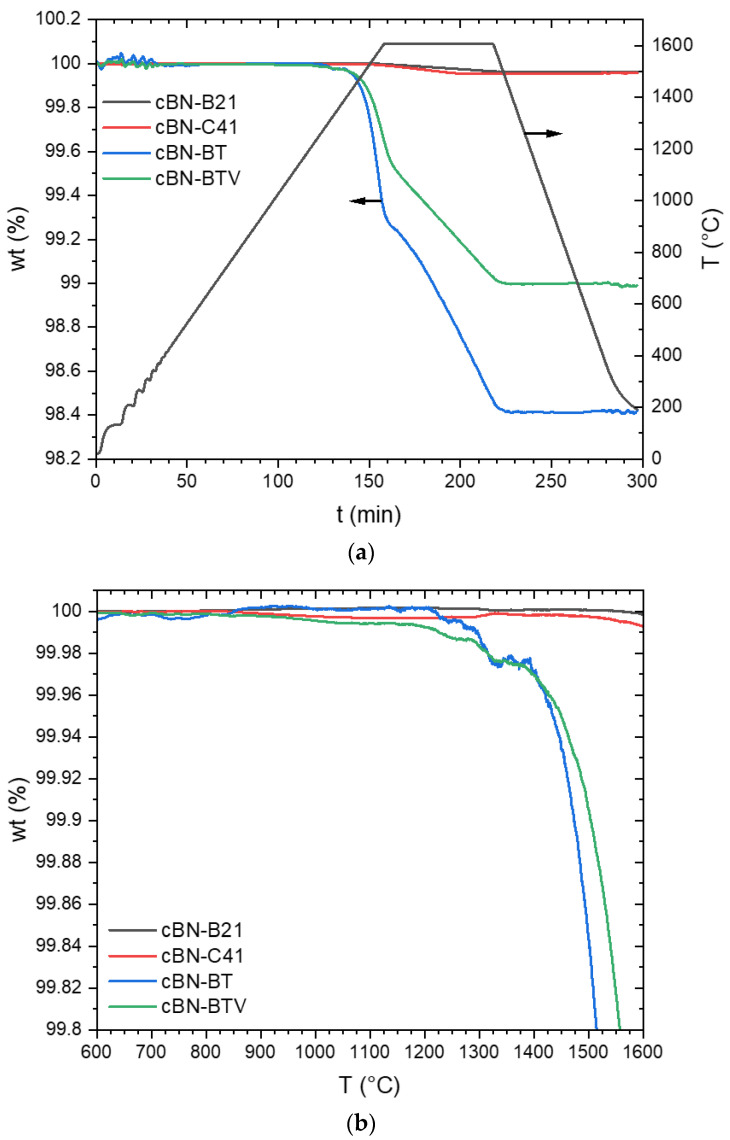
(**a**) Mass loss during the thermogravimetric analysis of the cBN powders B21, C41, BT and BTV under argon atmosphere and (**b**) mass loss as a function of temperature of the same (data of Figure 5a).

**Figure 6:**
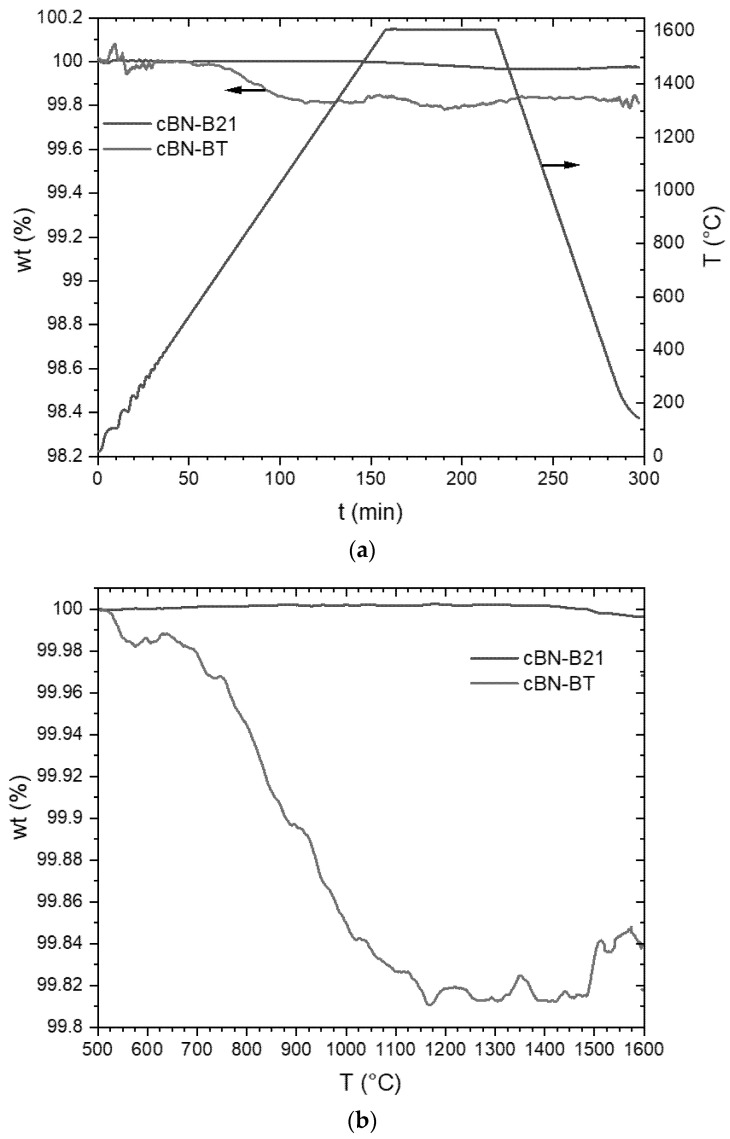
(**a**) Mass loss during thermogravimetric analysis under a nitrogen atmosphere of the cBN powders B21 und BT and (**b**) mass loss as a function of temperature of the same.

The results of the thermogravimetric analysis in argon atmosphere are given in Figure 5 and in nitrogen atmosphere in Figure 6. It must be considered that the absolute initial masses for the thermogravimetric investigation differs in a wide range (from approximately 180 mg to approximately 1000 mg). A lower initial mass leads to a higher relative noise of the mass change signal.

A significant loss of mass for the TiN coated cBN powders BT and BTV were observed during heat treatment under argon atmosphere (see also Table 2), whereas the uncoated powders B21 and C41 have a mass loss of only 0.04 wt. % (Figure 5a). In Figure 5b a small section of Figure 5a is plotted against temperature to show the onset of mass loss. The mass loss of the uncoated powders starts at 800 °C for C41 and at 1200 °C for B21. This mass loss is most probably caused by the evaporation of boric acid and B_2_O_3_ existing on the surface of the particles.

The mass change of the BT powder is small but complex at lower temperatures. This could be caused by the interaction with the still existing impurities on the surface. The BTV powder behaves like that of C41 at temperatures up to 1200 °C. However, the weight change in this temperature area is less than 0.01 wt. %. An acceleration of the weight loss starts for the coated powders BT and BTV at 1210 and 1175 °C respectively. This weight loss only stops at the end of the isothermal holding time. At the end of the holding time, the weight loss of the BTV powder is 1.58 wt. %, which is higher than for the BT powder (1.01 wt. %) (see Table 2).

A different behavior is observed in a nitrogen atmosphere (Figure 6a). The maximum mass loss for the TiN coated powder BT is only 0.17 wt. %, what is still slightly higher than for the uncoated powder B21 (0.02 wt. %). However, this is much less than for the coated BT powder tempered under argon (1.01 wt. %). Figure 6b also display the mass loss against temperature. It is obvious, that a very low loss of mass for the BT powder starts at 520 °C and for the uncoated B21 powder at 1400 °C.

**Figure 7:**
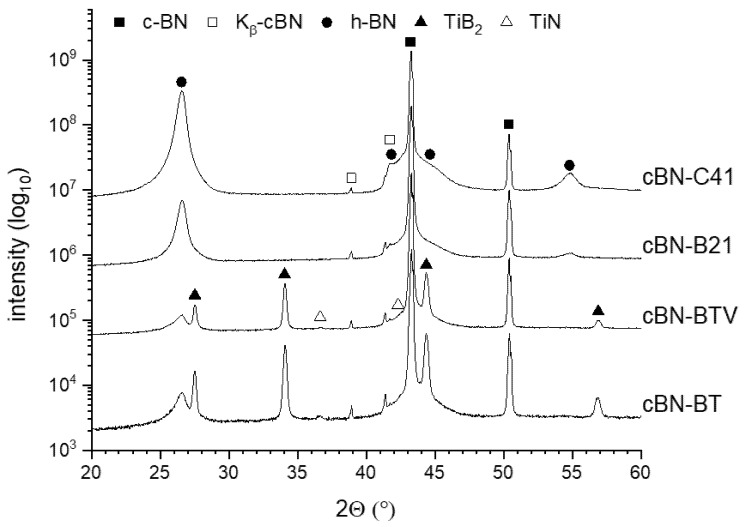
XRD patterns of the investigated cBN powders tempered at 1600 °C under an argon atmosphere (the logarithmic scaling emphasizes the minor phases with low peak intensity).

The results of the XRD analysis of the cBN powders tempered under argon at 1600 °C are represented in Figure 7. The cBN peaks (ICDD card: 00-035-1365) are clearly visible in all four powders as well as the broad peaks corresponding to the hBN modification (ICDD card: 01-085-1068). The hBN peaks of the uncoated powders (B21, C41) have a much higher intensity than those of the coated powders (BT, BTV), whereas the C41 powder exhibits the highest hBN amount. For the TiN coated powders, the TiN-phase observed in the initial state disappeared nearly completely after heat treatment. Obviously, it has converted into TiB_2_ (ICDD card: 01-085-2083).

**Figure 8:**
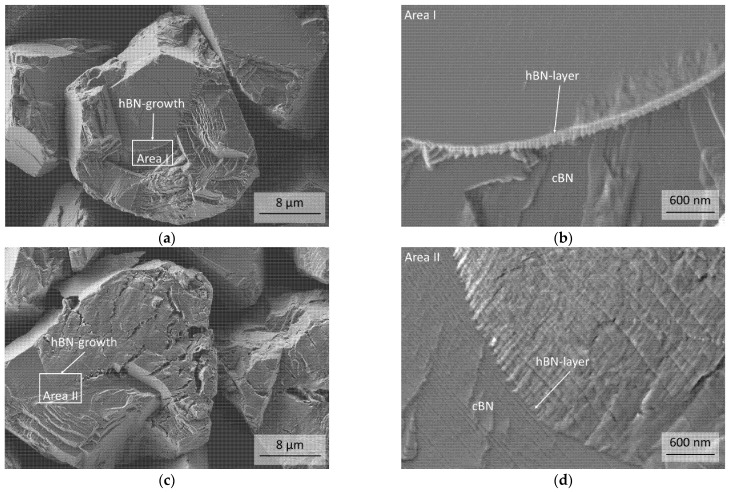
XRD patterns of the investigated cBN powders tempered at 1600 °C under nitrogen (the logarithmic scaling emphasizes the minor phases with low peak intensity).

The XRD patterns of the powders tempered under nitrogen shown in Figure 8 reveal the existence of cBN and hBN reflexes. However, the TiN phase (ICDD card: 01-087-0627) is stable under these conditions and no TiB_2_ phase was observed. The intensity of the formed hBN in the TiN coated cBN (BT) was lower compared to the uncoated powder B21.

**Figure 9:**
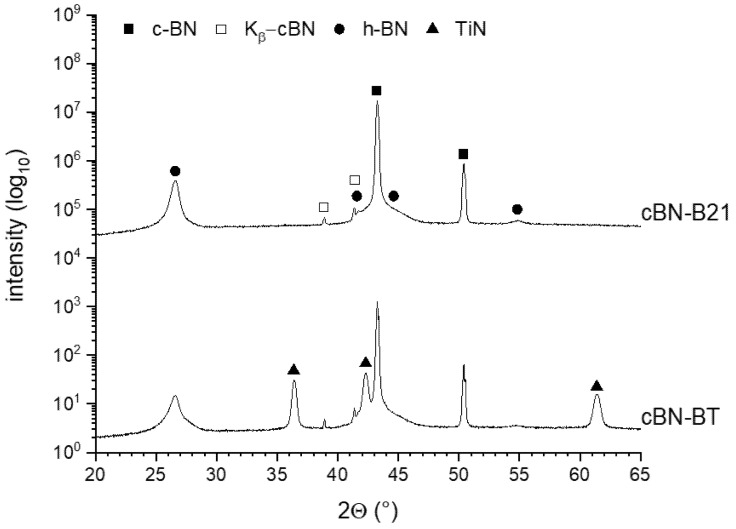
(**a**,**b**) FESEM micrographs of a particle of the under argon tempered C41 and (**c**,**d**) B21 powder with different magnifications.

The micrographs in Figure 9a–d show an overview of the surfaces of cBN particles of the uncoated C41 and B21 powder after the heat-treatment at 1600 °C. Detailed investigations show the begining of the formation of a hBN layer on the surface (Figure 9b,d). The growth starts at edges and steps and continues from there over the surface. Edges and steps are probably the preferred nucleation sites.

**Figure 10:**
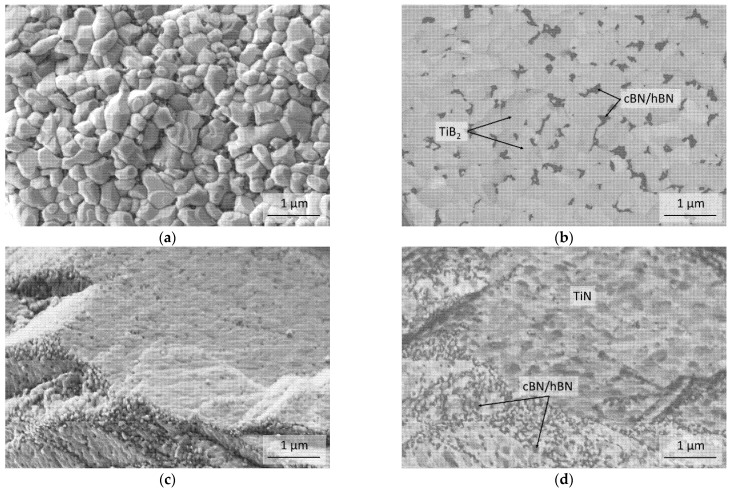
(**a**,**b**) FESEM micrographs of TiN coated cBN particles heat-treated at 1600 °C under argon and (**c**,**d**) under nitrogen (**a**,**c** with SE2 detector; **b**,**d** with ESB detector).

Micrographs of the particle surface of the coated BT powder heat-treated in argon and in nitrogen are shown in Figure 10. The surface looks different in comparison to the initial state (Figure 3). The Figure 10a,b micrographs prove that the TiN phase transformed during the heat-treatment under argon into the TiB_2_ phase, which exhibits a different grain shape. Because of the transformation, pores were formed within the coating in which hexagonal boron nitride can be found as it is shown in Figure 10b as dark grains. Due to this, the protection of the cBN particles by the coating cannot be achieved. 

However, Figure 10c,d shows that the TiN phase tempered under a nitrogen atmosphere also undergoes some recrystallization (compare Figure 3e). Additionally, for this coating due to the heat treatment, defects in the coating were formed. However, these are smaller and fewer in comparison with the Ar-treated powder. 

**Figure 11:**
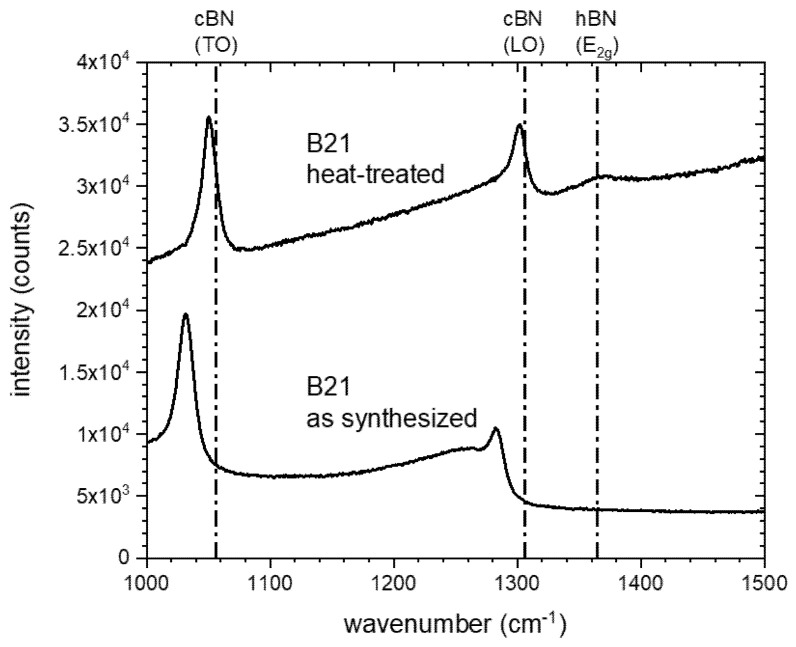
Raman spectra of the under argon tempered cBN powder B21 compared to the initial powder B21.

**Figure 12:**
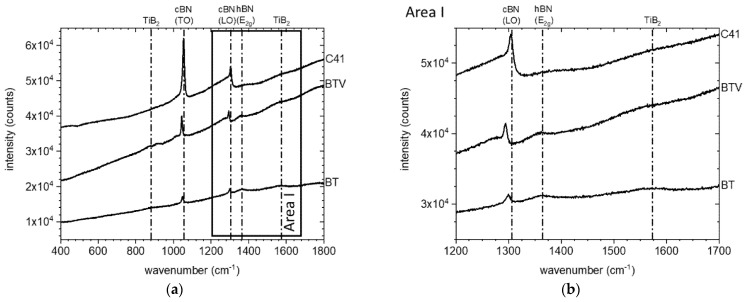

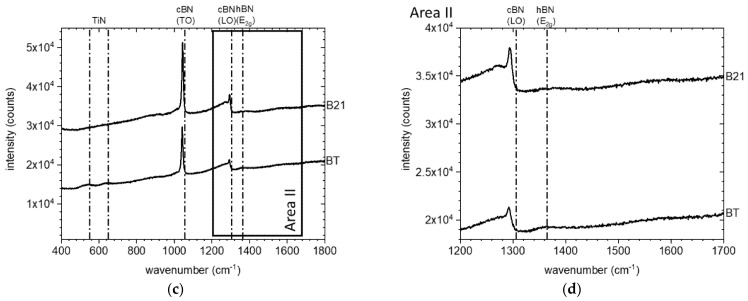
(**a**,**b**) Raman spectra of the heat-treated powders C41, BT and BTV under Ar atmosphere. (**c**,**d**) Raman spectra the heat-treated powders B21 and BT under N2 atmosphere. (**b**,**d**) are showing a detailed area of the hBN Raman bands in (**a**,**c**), respectively.

The results of the Raman investigations in Figure 11 and Figure 12 clearly reveal the Raman bands of cBN at 1056 and 1306 cm^−1^ [23]. The hBN band at 1365 cm^−1^ has a weak intensity in the heat-treated powder but the band is not observed in the starting powder. The Raman bands of the cBN in the heat-treated powder are shifted to lower wavenumbers. However, the starting powder has also a slight shift compared to the theoretical band positions. It probably has its origin in impurities [41] or/and residual stresses [42].

Figure 12a shows the Raman spectra of the at 1600 °C under Argon heat-treated powders C41, BTV and BT. The bands of TiB_2_ at 881 cm^−1^ [43] and 1573 cm^−1^ [44] are weak due to a low Raman activity and the low mass fraction of TiB_2_ in the powders BT and BTV. In all the heat-treated powders a similar weak hBN-band can be observed. 

The Raman spectra of heat-treated powders under N_2_ at 1600 °C are given in Figure 12c. In contrast to the TiN coated powders annealed under argon the BT powder exhibits Raman bands of TiN in a range of 400–650 cm^−1^ [45,46]. With a focus on the hBN formation, both variants do not show any significant difference in the hBN band (Figure 12b,d). 

## 4. Discussion

The XRD analysis in Figure 2 and the micrographs of the cross-sections of the TiN coated powders BT and BTV in Figure 4 prove a dense and homogenous TiN coating on the cBN particles. 

The higher weight loss during heat treatment in Ar of TiN coated powders BT and BTV in comparison to the uncoated powders (Figure 5 and Figure 6) is caused by the decomposition of the TiN coating. The XRD analysis (Figure 7) reveals the transformation of TiN into TiB_2_. Such a process does not take place during heat treatment in N_2_ (Figure 8). The micrographs in Figure 10 prove that the dense coating is decomposed and does not work as a protecting layer of the cBN grains.

The interaction of TiN with BN can be described by reaction (1) and (2):

2TiN+ 4cBN = 2TiB_2_ + 3N_2_ (1)

2TiN+ 4hBN = 2TiB_2_ + 3N_2_ (2)

**Figure 13:**
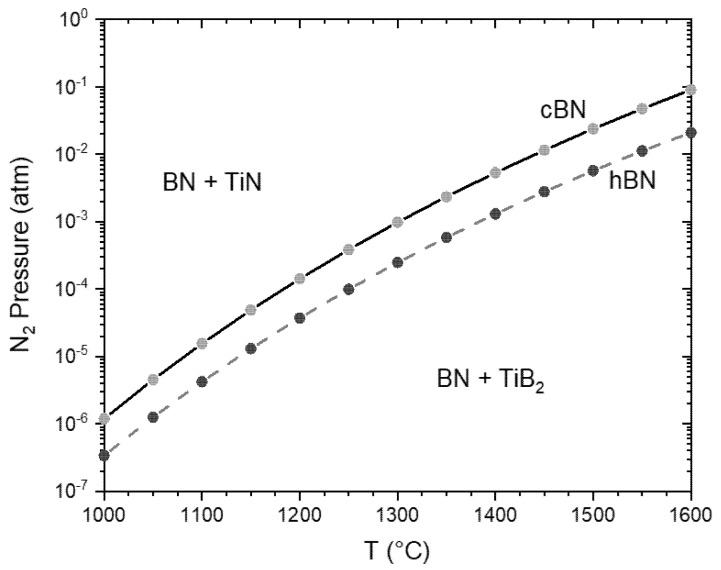
Nitrogen equilibrium pressure as a function of temperature for reaction (1) and (2). The stability area of the TiN and TiB_2_ phases are shown.

It is obvious that the reactions will result in a weight loss due to nitrogen release. Furthermore, it depends on the stability of TiN on the nitrogen pressure. The equilibrium nitrogen pressure for the cBN/TiN/TiB_2_ and the hBN/TiN/TiB_2_ phase mixture was calculated using FACTSAGE 8.0 (Figure 13). The database does not contain the data of cBN. The data in [1,23] for the phase transformation of c-BN «h-BN were used for the calculation. 

Above the lines in Figure 13, cBN or hBN is in equilibrium with TiN, below the lines with TiB_2_. This means if sintering or heat treatment takes place at nitrogen pressure below 2.9 × 10^−2^ atm at 1600 °C the TiN coating would decompose into TiB_2_ and nitrogen. The data predicts the instability of TiN/cBN for the heat treatment at 1600 °C in Ar and the stability of TiN/cBN in 1 atm nitrogen. This agrees with the experimental observed data of the TG–measurement and microstructural investigations.

**Figure 14:**
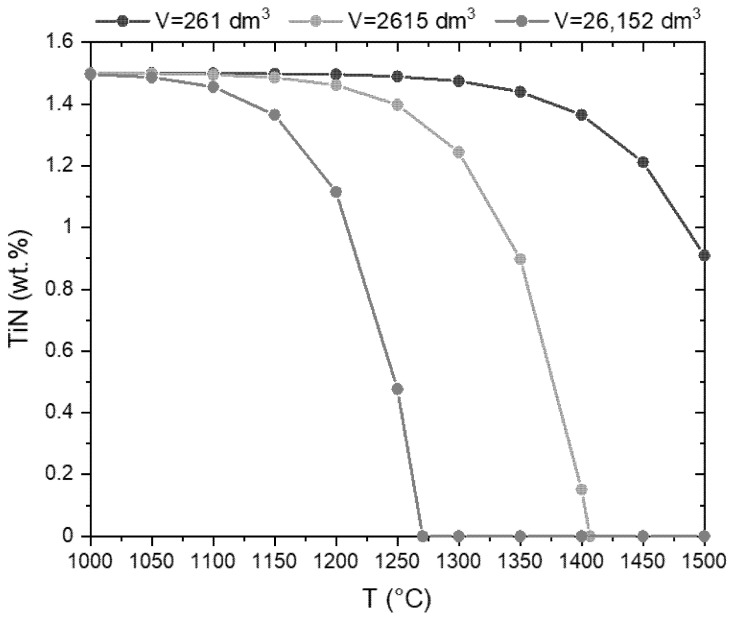
Decomposition of TiN in the mixture of (98.5 wt. % hBN and 1.5 wt. % TiN) according to reaction (2) as a function of temperature and the volume of the system (volume given at 1000 °C in (dm)^3^).

Based on the thermodynamics the decomposition of the TiN coating in Ar can take place even at low temperature. The TG measurements reveal a decomposition in argon only above approximately 1200 °C. This could have kinetic reasons and may be additionally caused by the formation of local nitrogen partial pressure due to the decomposition. This pressure could prevent or retard further reactions. This is shown exemplarily in Figure 14. The calculation was carried out for a mixture of 98.5 wt. % BN and 1.5 wt. % TiN with three different volumes of the system (defined by the Ar content). This could be a measure of the furnace volume or the gas flow rate multiplied by the time. The graphs show that in small volumes the decomposition is not complete even at 1500 °C, whereas a volume 100 times higher results in the complete decomposition at temperatures up to 1270 °C. On the other hand, the examples show that due to the low nitrogen equilibrium pressure decomposition of TiN practically does not take place at temperatures below 1050 °C.

The determined phase composition in the starting coated powders (see Table 1) can be used together with Equations (1) and (2) to calculate the mass loss during the heat treatment and to predict the amount of TiB_2_. The mass loss corresponds to the nitrogen release in Equations (1) and (2). The results of the calculations based on the TiN content measured by XRD are given in Table 3. The results are in good agreement with the observed weight losses and the measured amount of TiB_2_ in the heat-treated powder. This indicates that the interaction of TiN with BN is the main process responsible for the weight loss. Additionally, reactions with residual oxygen or moisture can play a role. However, in nitrogen with a similar purity, these effects result in a much lower weight loss. 

The investigations show that the TiN-coating on cBN particles could be a protective one only if a nitrogen partial pressure is formed on the surface. Otherwise, the formation of the TiB_2_ result in a destruction of the initially continuous TiN-coating.

However, very thin fine-grained TiN coatings, produced by ALD, could also undergo recrystallization at high temperatures in N_2_ despite the fact that they do not react with cBN. This recrystallization could also result in some damage to the coating (Figure 10d).

## 5. Conclusions

The investigation of the thermal stability of TiN coated cBN particles using thermogravimetric, phase and microstructural analysis reveal that,

- The stability of TiN coatings on cBN particles strongly depend on the nitrogen pressure. The decomposition into TiB_2_ was observed in Ar at temperatures above approximately 1200 °C. This reaction results in the formation of pores in the originally dense coating. Therefore, the layer could not more work as a protection layer.
- In a nitrogen atmosphere (1 atm pressure) no interaction of TiN with cBN was observed up to 1600 °C. However, the investigated very thin fine-grained TiN coatings, produced by ALD, showed after the heat treatment at 1600 °C a recrystallization, resulting also in very fine pores. Further investigations are necessary to clarify whether these processes also take place at 1300–1400 °C, at which composites are typically sintered with cBN. Additionally, the influence of the thickness and crystallite size of the coating needs further investigations.
- The observed results could be predicted by the thermodynamic calculations.

The results form a basis for the use of TiN-protective coatings to control the conversion of cBN in hBN accelerated by liquid phases during sintering of composites and for the optimization of the compaction processes. The coating is stable above temperatures of 1100–1200 °C only if a nitrogen partial pressure is higher than 1.5 × 10^−4^ atm or > 1 × 10^−3^ atm at 1300 °C (Figure 13). In molds of the SPS or hot presses with a small volume and a hindered gas exchange with the furnace chamber the decomposition of the TiN coating during densification can be stabilized by this reason. Additionally, a small amount of residual gas could have a stabilizing effect. 

However, further investigations have to be carried out to investigate the stability of the TiN coatings even in the thermodynamic stable region due to the fact, that nanocrystalline TiN-coatings can recrystallize during sintering or react with the matrix. At least, coatings with thicknesses up to 50 nm—produced by ALD and investigated here—were not stable at 1600 °C in nitrogen. The observed recrystallization resulted in small pores which might increase the conversion of cBN into hBN due to interaction with oxide liquids. Therefore, it is important to determine the necessary thickness and crystallite size of the TiN coating at the different sintering temperatures that are required to prevent cBN-hBN conversion.

The use of such coated cBN particles as abrasives or in composites additionally requires the investigation of the adhesion and wear resistance of the coated powders. These will be addressed as next steps.

## Figures and Tables

**Table 1:** Properties of the used cubic boron nitride (cBN) powders and TiN coated cBN powders regarding oxygen content, metallic impurities, coating content and coating thickness (the TiN content was determined by XRF measurement of Ti and XRD).

cBN-Powder	Oxygen Content (wt. %)	Metallic Impurities (wt. %)				TiN-Coating Content (XRF/XRD) (wt. %)	TiN-Coating Thickness (nm)
		Fe	Si	Cl	Cr		
B20	0.122 ± 0.042	0.0028	0.03	-	-	-	-
B21	0.101 ± 0.015	0.0027	0.02	-	-	-	-
C41	0.113 ± 0.023	0.0028	0.02	-	-	-	-
BT	0.170 ± 0.023	0.02	0.03	0.05	0.0085	2.3/2.3 ± 0.1	50
BTV	0.135 ± 0.032	0.01	0.03	0.02	0.0080	1.4/1.3 ± 0.1	20

**Table 2:** Mass losses of the cBN-powders after thermogravimetric analysis under argon and nitrogen atmosphere up to a temperature of 1600 °C.

cBN-Powder	Coating	Atmosphere	Mass Loss (wt. %)
B21	-	argon	0.04
C41	-		0.04
BT	TiN		1.58
BTV	TiN		1.01
B21	-	nitrogen	0.02
BT	TiN		0.17

**Table 3:** By XRD analysis measured and calculated phase contents of TiN and TiB_2_ in the initial and in the Ar heat-treated cBN powders and measured and calculated weight loss during heat treatment according to reaction (1) and (2).

TiN Coated cBN-Powder	Measured TiN Content before TGA (wt. %)	Measured TiB_2_ Content after TGA (wt. %)	Calculated TiB_2_ Content (wt. %)	Calculated Weight Loss (wt. %)	Loss of Mass after TGA (wt. %)
BT	2.3 ± 0.1	2.8 ± 0.1	2.6	1.56	1.58
BTV	1.3 ± 0.1	1.8 ± 0.1	1.5	0.88	1.01

## Data Availability

The data presented in this study are available on request from the corresponding author.
